# 

**Published:** 1895-05-04

**Authors:** 


### CONTENTS.

Damaged Lives and Assurance Medicine - - - 73

Opium in India - - - - - - - - - - 74

On Modern Progress in Ophthalmic Medicine and Surgery. By R. BRUDENELL CARTER, F.R.C.S. - - - - 75

Modern Sociology.—Brothers in Affliction - - - - - 76

Medical Progress and Hospital Clinics—

The Royal Commission on Tuberculosis - - - - - - 79

On the Causes and Treatment of Colds in the Head of Influenzal Origin - - - - - - - - - - - 81

PROGRESS IN MEDICINE.—Renal Medicine - - - - - 81

PROGRESS IN SURGERY.—Surgery of the Intestines - - - 83

NEW APPLIANCES AND THINGS MEDICAL - - - - - 84

Annotations.—Private Hospitals in Dublin—The Outcry Against Pay Wards—The Farmyard Pump - - - - - - - 77

Notes and News - - - - - - - - - - - - 78

Editor's Letter-Box - - - - - - - - - - - 84

The Institutional Workshop:

PRACTICAL DEPARTMENTS.—Ward Fittings at the London Temperance Hospital - - - - - - - - - - 85

HOSPITAL FESTIVALS AND MEETINGS - - - - - - 86

PRACTICAL POINTS - - - - - - - - - - 86

Around the Hospitals - - - - - - - - - - 86

Scraps and Gleanings - - - - - - - - - - 86

The Book World of Medicine and Science - - - - 87

### EXTRA SUPPLEMENT.—THE NURSING MIRROR.

News from the Nursing World.—Royal Inspections—Queen Victoria's Jubilee Institute—Certificated Midwives—Holidays Well Earned—North London Hospital for Consumption—Work for Women—A Lecture for Nurses—A Nameless Nurse—Women Lecturers—Nurses at Dundee—Is Bombay Philanthropic?—Short Items - - - - - - xxix

Elementary Anatomy and Surgery for Nurses. By W. MCADAM ECCLES, M.B., M.S., F.R.C.S. XIV.—The Circulatory System - - - - - - - - - - xxxi

Nursing in America.—The Convention of Superintendents and Members of the Association - - - - - - xxxii

A Nurse at Aden - - - - - - - - - - xxxii

Letters from Upper Burma. By MRS. ERNEST HART - xxxiii

The Nursing World - - - - - - - - - - xxxiii

Invalid Children's Aid Association - - - - - xxxiii

French Schools for Trained Nurses: Their Origin and Organization - - - - - - - - - - xxxiv

Novelties for Nurses - - - - - - - - - xxxiv

The Great Lahore Durbar of 1894 - - - - - - xxxv

Notes and Queries—Where to Go - - - - - - xxxvi

Everybody's Opinion - - - - - - - - - xxxvii

Registration of Midwives - - - - - - - - xxxvii

Appointments - - - - - - - - - - - xxxvii

The Volunteer Medical Staff Corps at Netley - - xxxviii

